# Supplementary material for: Tetraspanins are unevenly distributed across single extracellular vesicles and bias sensitivity to multiplexed cancer biomarkers
Source: J Nanobiotechnology. 2021 Aug 21;19:250. doi: 10.1186/s12951-021-00987-1 (PMC8379740; doi:10.1186/s12951-021-00987-1)
Supplement: Supplementary file 1 — Additional file 1: Figure S1. EV sizing by TEM. At least 50 particles were analyzed in 3 each of 3 images taken of SK-OV-3 EVs isolated by UC. Diameter of individual particles was estimated in the Fiji package of ImageJ by circling deflated EVs, as seen by thin white lines overlayed on TEM images. Figure S2. SK-OV-3 EVs isolated from separate bioreactors. The tetraspanin profile of EVs from a first bioreactor (a), copied from Fig. 3 for comparison, is similar to the tetraspanin profile of SK-OV-3 EVs isolated from a second bioreactor (b). Although there is a difference in CD81 capture/CD81 detection, this change was common between consecutive weeks from a single bioreactor. Figure S3. FCMPASS Refractive Index Parameters and Modeling. Using scatter of a variety of standard beads, the size of SK-OV-3 EVs was modeled using (a) these parameters for average EV refractive index and membrane thickness. (b) FCMPASS model of SSC-H vs. diameter of EVs by measuring polystyrene bead SSC-H of varying sizes. Figure S4. Gating scheme for tetraspanin analysis. In row 1, EVs are gated from background noise by examining PBS versus an EV only control. In row 2, gates were used to separate the real events from those at the limits of fluorescence (at 0 fluorescence) to find the median fluorescent intensity of the EVs. In row 3, fluorescence-minus-one controls were used to gate fluorescent particles from unstained particles. In row 4 these gates were adjusted to encompass no more than ~ 5% of the antibody aggregates. In row 5, fully stained EVs are shown as an example of where these gates appeared on fluorescently labeled particles. Figure S5. Flow cytometry of EVs from various sources labeled with anti-tetraspanin antibodies. SSC-A vs (a) anti-CD9-CF488, (b) anti-CD63-CF647, and (c) anti-CD81-CF555 of control samples and labeled EVs. EV only sample is SK-OV-3 EVs. Figure S6. Controls show that tetraspanin expression profile is not biased by presence of bovine EVs from FBS or by small di [file 12951_2021_987_MOESM1_ESM.docx]

**Additional file 1 for**

Tetraspanins are unevenly distributed across single extracellular vesicles and bias sensitivity to multiplexed cancer biomarkers

Rachel R. Mizenko^1^, Terza Brostoff^2^, Tatu Rojalin^1^, Hanna J. Koster^1^, Hila S. Swindell^3^, Gary S. Leiserowitz^4^, Aijun Wang^1,3^, Randy P. Carney^1^

^1^Department of Biomedical Engineering, University of California, Davis, United States

^2^Department of Pathology, University of California, San Diego, United States

^3^Department of Surgery, University of California, Davis, United States

^4^Division of Gynecologic Oncology, University of California Davis Medical Center, Sacramento, California, United States


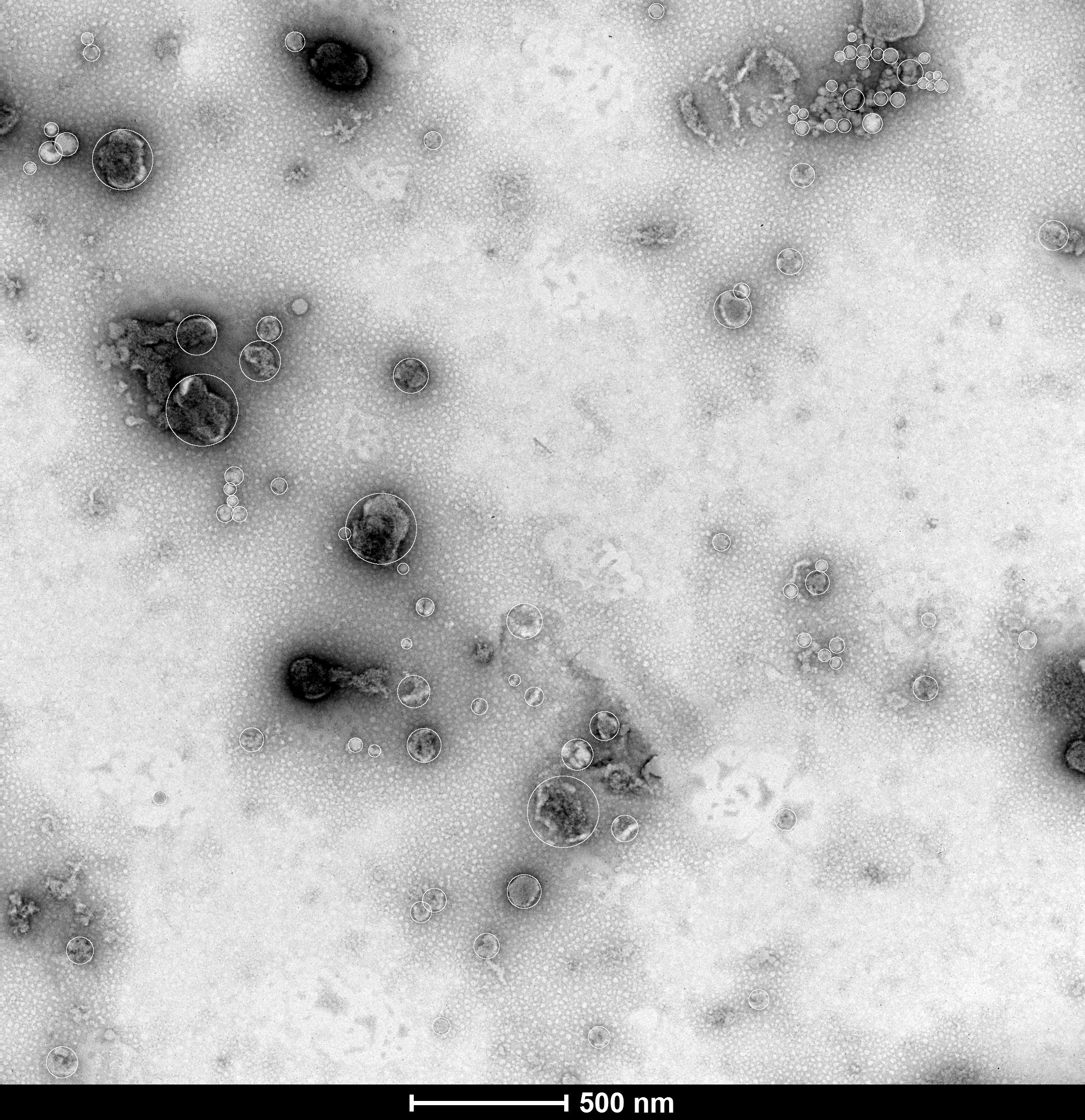

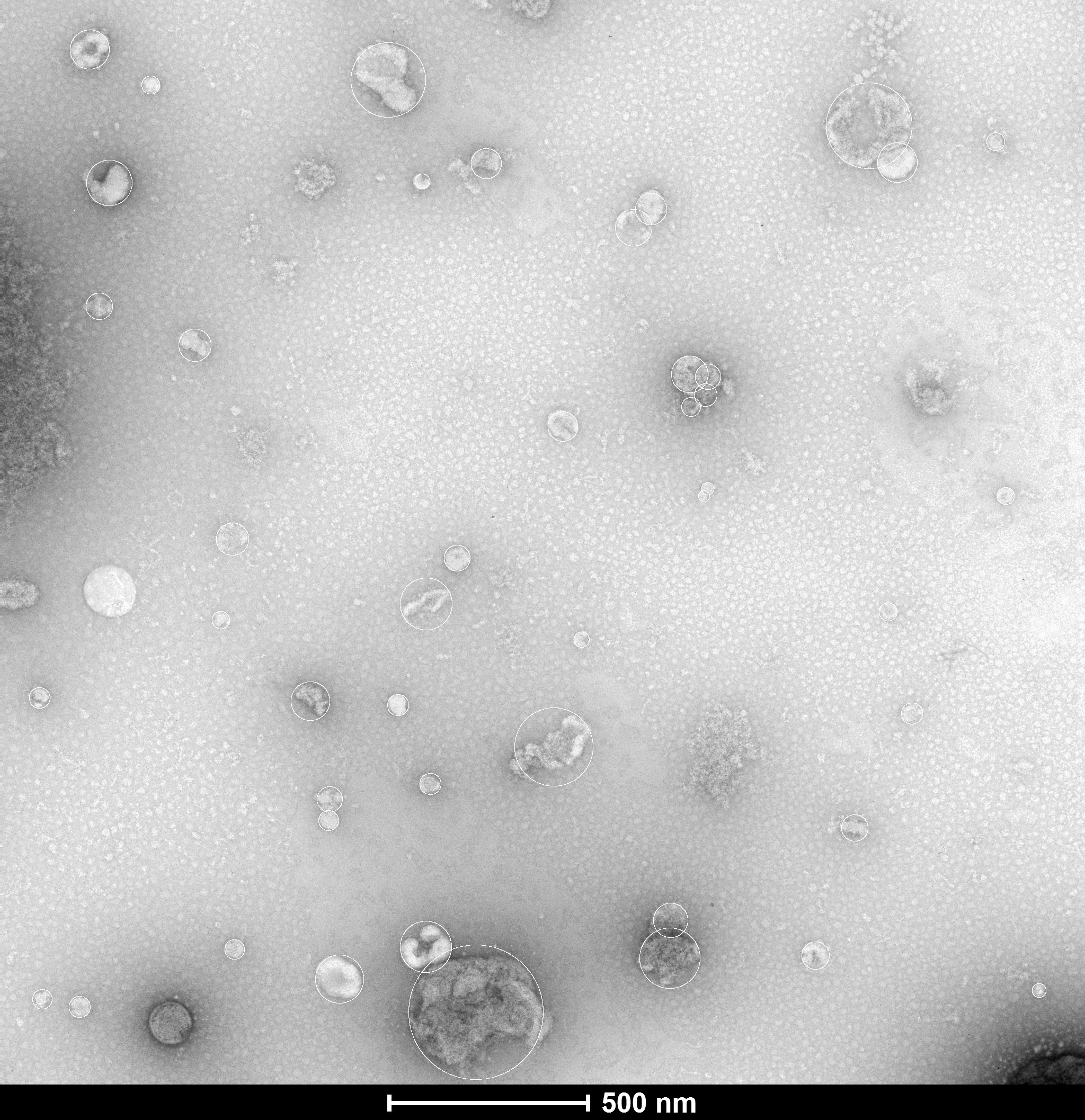

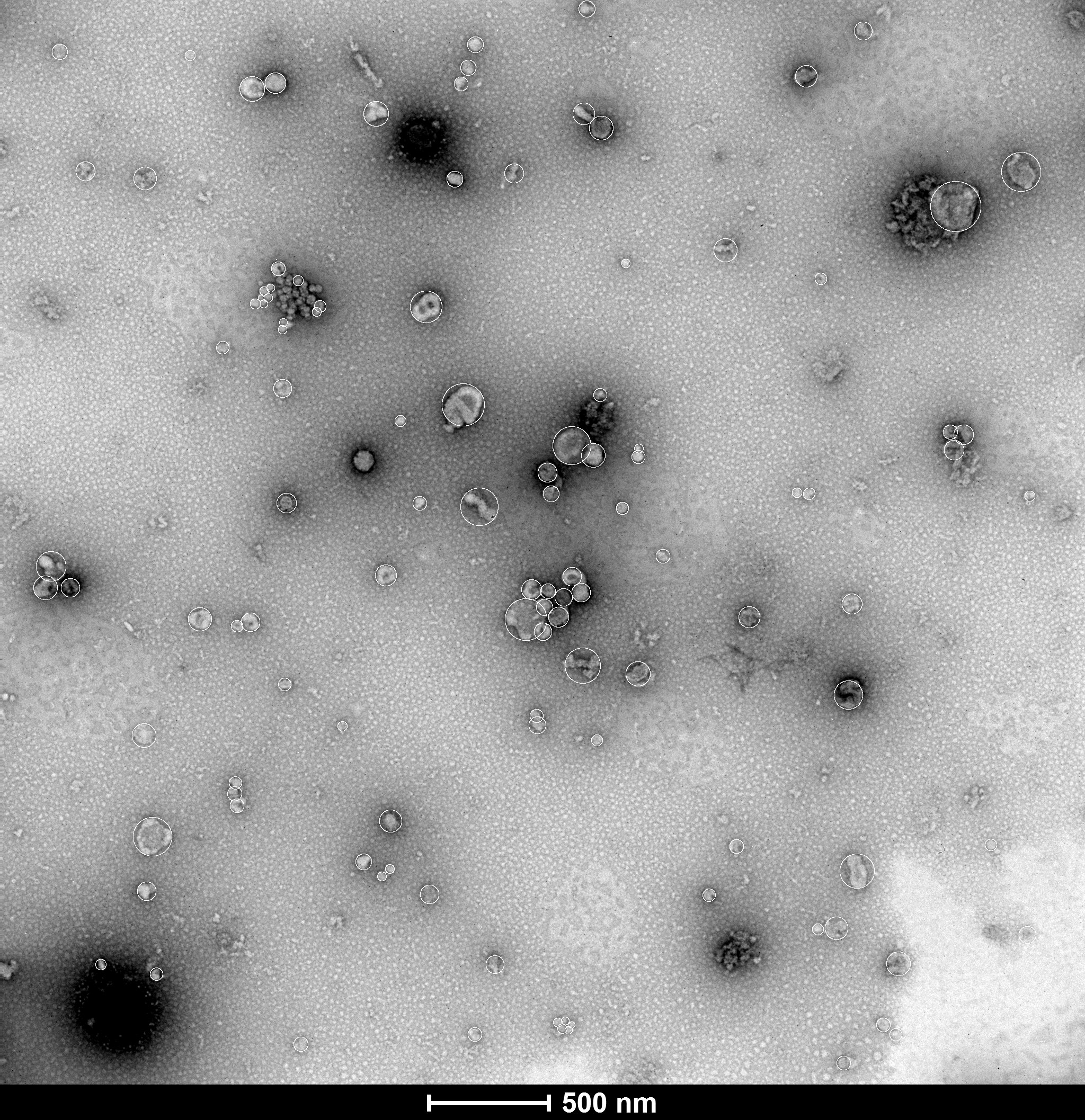


**Figure S1:** EV sizing by TEM. At least 50 particles were analyzed in 3 each of 3 images taken of SK-OV-3 EVs isolated by UC. Diameter of individual particles was estimated in the Fiji package of ImageJ by circling deflated EVs, as seen by thin white lines overlayed on TEM images.

­­


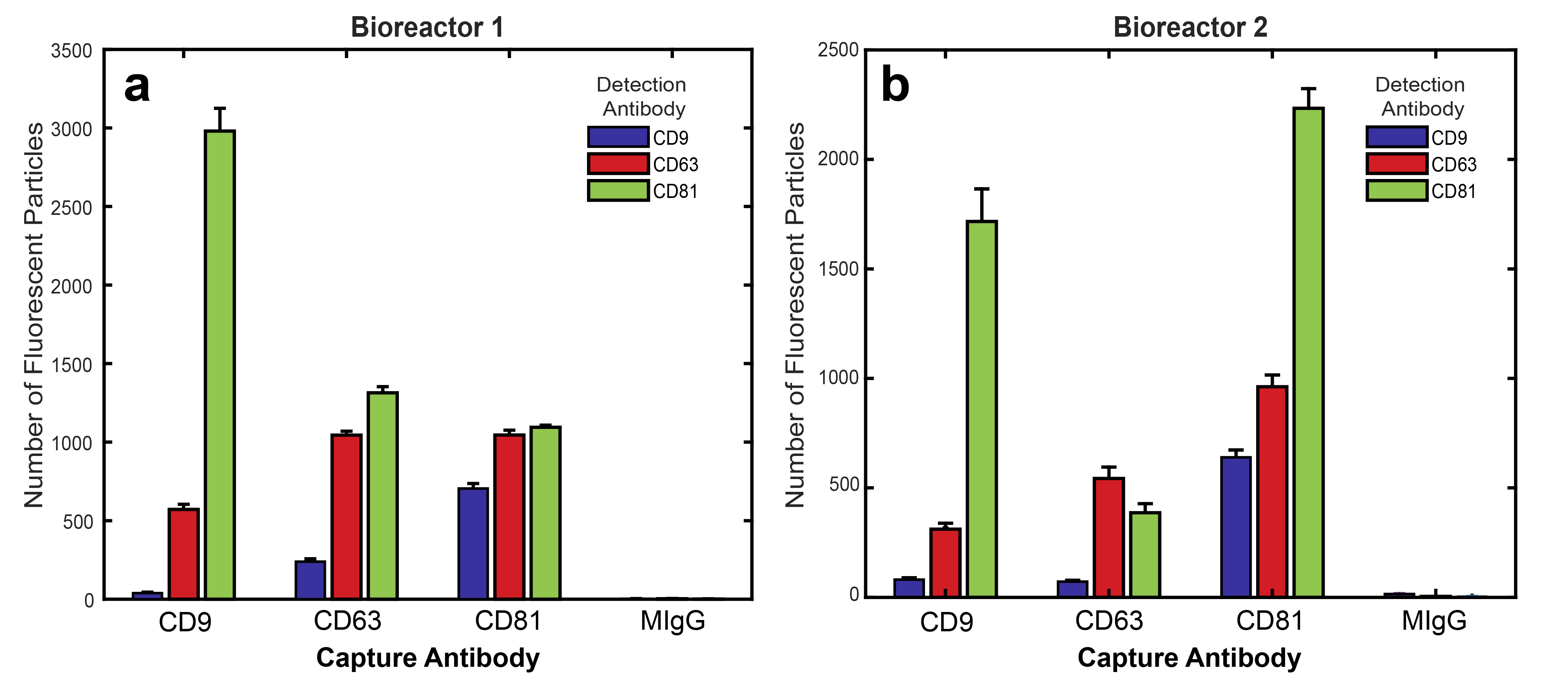


**Figure S2:** SK-OV-3 EVs isolated from separate bioreactors. The tetraspanin profile of EVs from a first bioreactor (a), copied from Figure 3 for comparison, is similar to the tetraspanin profile of SK-OV-3 EVs isolated from a second bioreactor (b). Although there is a difference in CD81 capture/CD81 detection, this change was common between consecutive weeks from a single bioreactor.

**
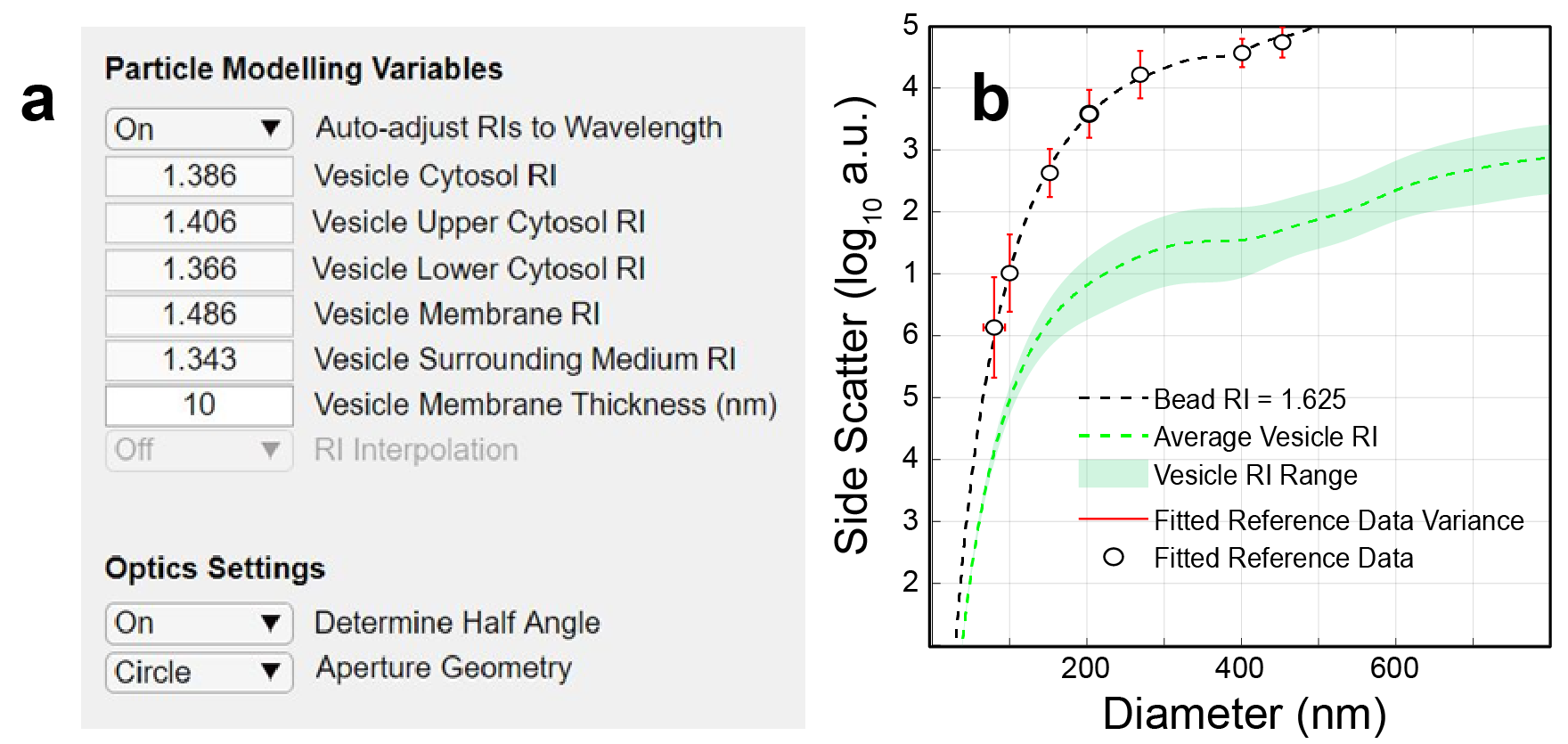
**

**Figure S3:** FCM_PASS_ Refractive Index Parameters and Modeling. Using scatter of a variety of standard beads, the size of SK-OV-3 EVs was modeled using (a) these parameters for average EV refractive index and membrane thickness. (b) FCM_PASS_ model of SSC-H vs. diameter of EVs by measuring polystyrene bead SSC-H of varying sizes.


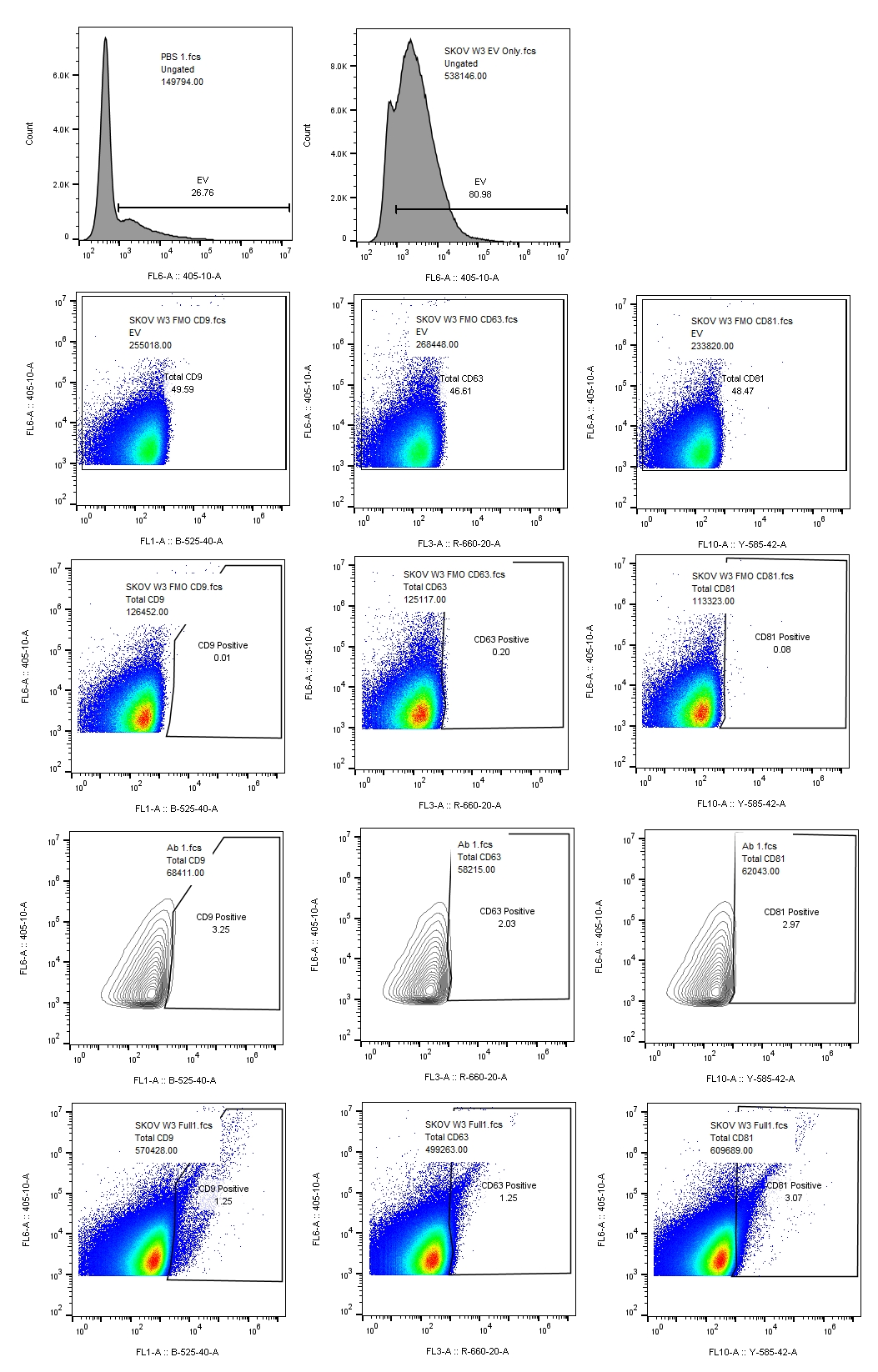


**Figure S4:** Gating scheme for tetraspanin analysis. In row 1, EVs are gated from background noise by examining PBS versus an EV only control. In row 2, gates were used to separate the real events from those at the limits of fluorescence (at 0 fluorescence) to find the median fluorescent intensity of the EVs. In row 3, fluorescence-minus-one controls were used to gate fluorescent particles from unstained particles. In row 4 these gates were adjusted to encompass no more than ~5% of the antibody aggregates. In row 5, fully stained EVs are shown as an example of where these gates appeared on fluorescently labeled particles.

Flow cytometry of EVs from various sources labeled with anti-tetraspanin antibodies. SSC-A vs (a) anti-CD9-CF488, (b) anti-CD63-CF647, and (c) anti-CD81-CF555 of control samples and labeled EVs. EV only sample is SK-OV-3 EVs.

**
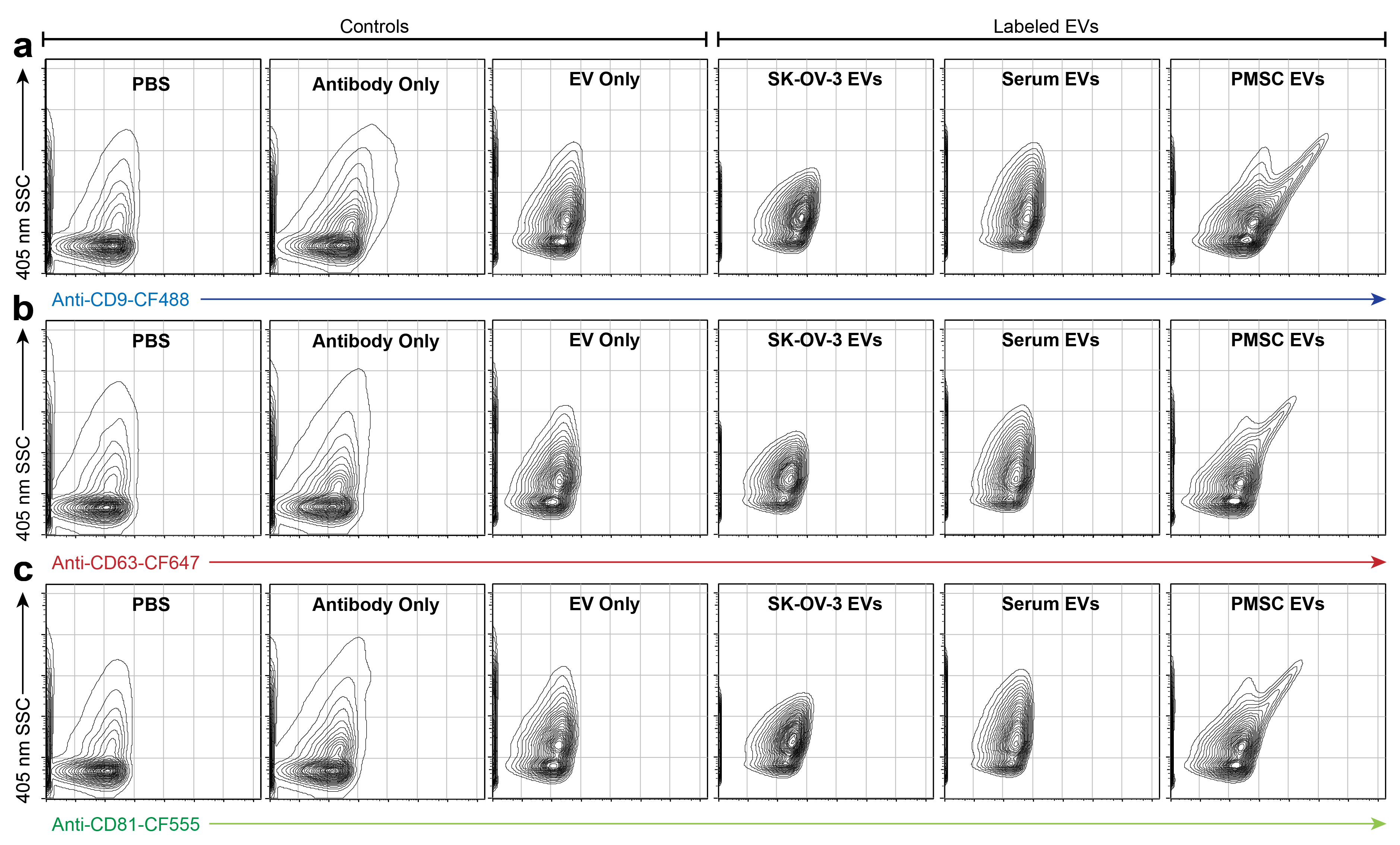
Figure S5:** Flow cytometry of EVs from various sources labeled with anti-tetraspanin antibodies. SSC-A vs (a) anti-CD9-CF488, (b) anti-CD63-CF647, and (c) anti-CD81-CF555 of control samples and labeled EVs. EV only sample is SK-OV-3 EVs.

**
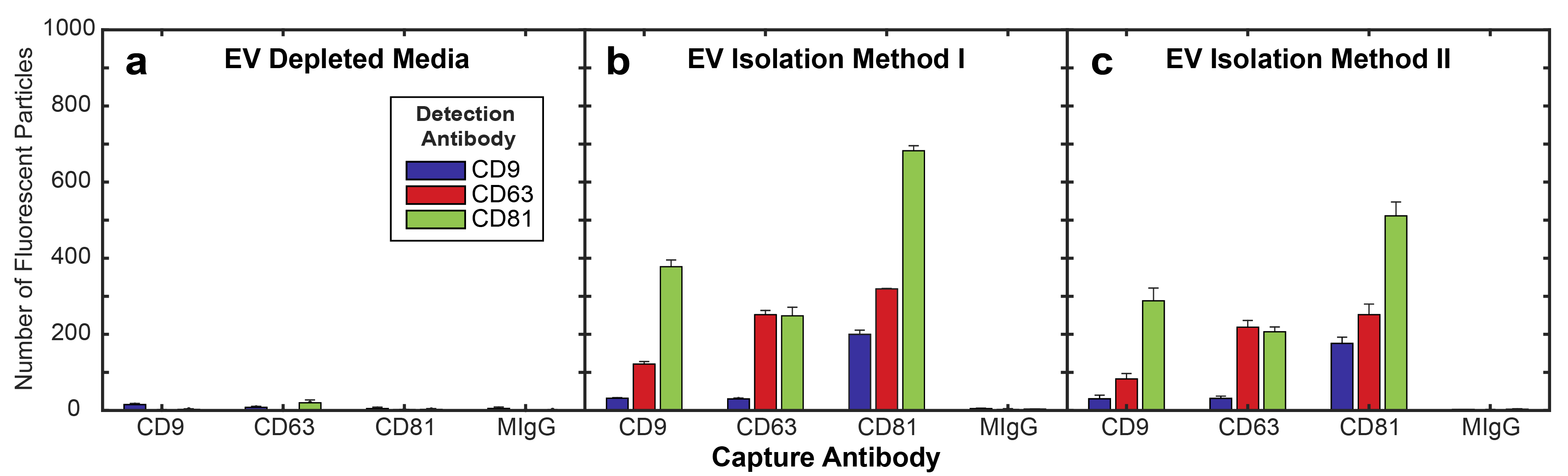
**

**Figure S6:** Controls show that tetraspanin expression profile is not biased by presence of bovine EVs from FBS or by small differences in ultracentrifugation isolation procedure. (a) McCoy’s 5A, 1X (Iwakata and Grad Mod.) with L-glutamine supplemented with 10% FBS EV depleted overnight at 100,000xg and (b) SK-OV-3 conditioned media were subjected to the same UC Method I isolation procedure. (c) The same volume of SK-OV-3 conditioned media was subjected to UC Method II isolation procedure. All samples were diluted 500x prior to completing tetraspanin analysis on the ExoView. (a) EV-depleted media showed relatively few detected EVs while both isolation methods had high numbers of EVs with similar patterns of tetraspanin expression.
